# 

**Published:** 1894-09

**Authors:** 


					﻿THE
Southern Medical Record.
A Monthly Journal of Medicine and Surgery.
Vol. XXIV. ATLANTA, GA., SEPTEMBER, 1894.	No. 9>
EDITORS :
A. w. GRIGGS, M. D.	WILLIS F. WESTMORELAND, M. D.
j. McFadden gaston, m. d.	louis h. jones, m. d.
DAN. H. HOWELL, M. D., Business Manager.
Entered at the Postotttce at Atlanta, Ga., as Second-Class Matter.
The Franklin Printing and Publishing Jo., Atlanta, Ga.
				

